# Comparison of fresh testicular sperm aspiration and use of either thawed pre-frozen sperm or oocyte freezing: impact on cumulative live birth rates for couples experiencing ejaculation failure

**DOI:** 10.1093/humrep/deae290

**Published:** 2024-12-31

**Authors:** Shaoquan Zhan, Geng An, Jiayu Gan, Hongzi Du, Xin Fu, Chunyan Wang, Yuling Mao, Xiangjin Kang, Jianqiao Liu, Hanyan Liu

**Affiliations:** Department of Obstetrics and Gynecology, Center for Reproductive Medicine, Guangdong Provincial Key Laboratory of Major Obstetric Diseases, Guangdong Provincial Clinical Research Center for Obstetrics and Gynecology, Guangdong-Hong Kong-Macao Greater Bay Area Higher Education Joint Laboratory of Maternal-Fetal Medicine, The Third Affiliated Hospital of Guangzhou Medical University, Guangzhou, China; Key Laboratory for Reproductive Medicine of Guangdong Province, The Third Affiliated Hospital of Guangzhou Medical University, Guangzhou, China; Department of Obstetrics and Gynecology, Center for Reproductive Medicine, Guangdong Provincial Key Laboratory of Major Obstetric Diseases, Guangdong Provincial Clinical Research Center for Obstetrics and Gynecology, Guangdong-Hong Kong-Macao Greater Bay Area Higher Education Joint Laboratory of Maternal-Fetal Medicine, The Third Affiliated Hospital of Guangzhou Medical University, Guangzhou, China; Key Laboratory for Reproductive Medicine of Guangdong Province, The Third Affiliated Hospital of Guangzhou Medical University, Guangzhou, China; Department of Obstetrics and Gynecology, Center for Reproductive Medicine, Guangdong Provincial Key Laboratory of Major Obstetric Diseases, Guangdong Provincial Clinical Research Center for Obstetrics and Gynecology, Guangdong-Hong Kong-Macao Greater Bay Area Higher Education Joint Laboratory of Maternal-Fetal Medicine, The Third Affiliated Hospital of Guangzhou Medical University, Guangzhou, China; Department of Obstetrics and Gynecology, Center for Reproductive Medicine, Guangdong Provincial Key Laboratory of Major Obstetric Diseases, Guangdong Provincial Clinical Research Center for Obstetrics and Gynecology, Guangdong-Hong Kong-Macao Greater Bay Area Higher Education Joint Laboratory of Maternal-Fetal Medicine, The Third Affiliated Hospital of Guangzhou Medical University, Guangzhou, China; Key Laboratory for Reproductive Medicine of Guangdong Province, The Third Affiliated Hospital of Guangzhou Medical University, Guangzhou, China; Department of Obstetrics and Gynecology, Center for Reproductive Medicine, Guangdong Provincial Key Laboratory of Major Obstetric Diseases, Guangdong Provincial Clinical Research Center for Obstetrics and Gynecology, Guangdong-Hong Kong-Macao Greater Bay Area Higher Education Joint Laboratory of Maternal-Fetal Medicine, The Third Affiliated Hospital of Guangzhou Medical University, Guangzhou, China; Department of Obstetrics and Gynecology, Center for Reproductive Medicine, Guangdong Provincial Key Laboratory of Major Obstetric Diseases, Guangdong Provincial Clinical Research Center for Obstetrics and Gynecology, Guangdong-Hong Kong-Macao Greater Bay Area Higher Education Joint Laboratory of Maternal-Fetal Medicine, The Third Affiliated Hospital of Guangzhou Medical University, Guangzhou, China; Department of Obstetrics and Gynecology, Center for Reproductive Medicine, Guangdong Provincial Key Laboratory of Major Obstetric Diseases, Guangdong Provincial Clinical Research Center for Obstetrics and Gynecology, Guangdong-Hong Kong-Macao Greater Bay Area Higher Education Joint Laboratory of Maternal-Fetal Medicine, The Third Affiliated Hospital of Guangzhou Medical University, Guangzhou, China; Department of Obstetrics and Gynecology, Center for Reproductive Medicine, Guangdong Provincial Key Laboratory of Major Obstetric Diseases, Guangdong Provincial Clinical Research Center for Obstetrics and Gynecology, Guangdong-Hong Kong-Macao Greater Bay Area Higher Education Joint Laboratory of Maternal-Fetal Medicine, The Third Affiliated Hospital of Guangzhou Medical University, Guangzhou, China; Department of Obstetrics and Gynecology, Center for Reproductive Medicine, Guangdong Provincial Key Laboratory of Major Obstetric Diseases, Guangdong Provincial Clinical Research Center for Obstetrics and Gynecology, Guangdong-Hong Kong-Macao Greater Bay Area Higher Education Joint Laboratory of Maternal-Fetal Medicine, The Third Affiliated Hospital of Guangzhou Medical University, Guangzhou, China; Key Laboratory for Reproductive Medicine of Guangdong Province, The Third Affiliated Hospital of Guangzhou Medical University, Guangzhou, China; Department of Obstetrics and Gynecology, Center for Reproductive Medicine, Guangdong Provincial Key Laboratory of Major Obstetric Diseases, Guangdong Provincial Clinical Research Center for Obstetrics and Gynecology, Guangdong-Hong Kong-Macao Greater Bay Area Higher Education Joint Laboratory of Maternal-Fetal Medicine, The Third Affiliated Hospital of Guangzhou Medical University, Guangzhou, China; Key Laboratory for Reproductive Medicine of Guangdong Province, The Third Affiliated Hospital of Guangzhou Medical University, Guangzhou, China

**Keywords:** ejaculatory failure, oocyte vitrification, frozen sperm, testicular sperm aspiration (TESA), oocyte retrieval, IVF, cumulative live birth

## Abstract

**STUDY QUESTION:**

Is there a difference in the cumulative live birth rate (CLBR) after fresh testicular sperm aspiration (TESA) compared with the use of either pre-frozen sperm or oocyte freezing for couples experiencing ejaculation failure on the day of oocyte retrieval?

**SUMMARY ANSWER:**

After adjusting for confounding factors, the use of pre-frozen sperm or the freezing and thawing of oocytes appeared to be as effective as TESA in achieving CLBRs for couples experiencing temporary ejaculation failure.

**WHAT IS KNOWN ALREADY:**

Male patients may be concerned about experiencing temporary ejaculation failure on the day of their partner’s oocyte retrieval, in which case they may choose surgical sperm retrieval, oocyte freezing on the day, or have their sperm frozen in advance. However, the clinical efficacy of these three options has not yet been evaluated.

**STUDY DESIGN, SIZE, DURATION:**

A retrospective data analysis was conducted on 65 178 oocyte retrieval cycles at a university-affiliated IVF center from January 2012 to May 2021.

**PARTICIPANTS/MATERIALS, SETTING, METHODS:**

The overall characteristics, completed cycle characteristics, and clinical outcomes were analyzed among couples with ejaculation failure who underwent three distinct clinical interventions, with those receiving TESA serving as the control group. The primary outcome measure was the CLBR, and the secondary outcome measures were the clinical pregnancy rate (CPR) and live birth rate (LBR) per embryo transfer. A robust (modified) Poisson regression model was used to evaluate the association between the three clinical options for ejaculation failure and CLBRs.

**MAIN RESULTS AND ROLE OF CHANCE:**

Of the eligible oocyte retrieval cycles, 756 cycles (1.2%) experienced ejaculation failure, with 640 cycles completing treatment. These treatments included 325 cycles using TESA, 227 cycles utilizing pre-frozen sperm, and 88 cycles involving frozen–thawed oocytes. The CLBRs for the TESA, thawed-sperm and thawed-oocyte groups were 36.9%, 48.9%, and 34.1%, respectively, showing a statistically significant difference (*P* = 0.007). Specifically, the thawed-sperm group demonstrated a significantly higher CLBR compared to the TESA group, while no significant difference was observed between the TESA and thawed-oocyte groups. Similarly, the CPRs and LBRs per embryo transfer for the three groups were 37.4%, 50.0%, and 41.8%, respectively (*P* = 0.005), and 29.9%, 39.6%, and 33.0%, respectively (*P* = 0.030). Again, the thawed-sperm group showed a significantly higher CPR and a significantly higher LBR, but no significant differences for the thawed-oocyte group, compared to the TESA group. Notably, the significant differences in both CLBR and LBR emerged after the second embryo transfer. However, after adjusting for multiple factors, including female age at oocyte retrieval, type and duration of infertility, female body mass index, number of previous IVF cycles, ovarian stimulation protocol, endometrial thickness on the last ultrasound, insemination method, number of oocytes retrieved, number of fertilized oocytes, and number of usable embryos on Day 3, the analysis revealed no significant association between CLBR and the use of pre-frozen sperm (risk ratio (RR) 1.08, 95% confidence interval (CI) 0.81–1.44) or thawed oocytes (RR 1.01, 95% CI 0.76–1.33), compared with TESA.

**LIMITATIONS, REASONS FOR CAUTION:**

Given that the study is retrospective and the sample size is too small, particularly concerning the use of thawed oocytes, we acknowledge that the data present here is only suggestive and refers to an association that warrants cautious interpretation. Therefore, further research in the form of prospective studies as well as randomized controlled trials is needed to provide a definitive answer to the research question.

**WIDER IMPLICATIONS OF THE FINDINGS:**

Our findings suggest that using pre-frozen sperm or frozen-thawed oocytes can offer comparable CLBRs to TESA for cases of temporary ejaculation failure, providing clinical alternatives that may reduce the logistical challenges in ART cycles.

**STUDY FUNDING/COMPETING INTEREST(S):**

This study was supported by the National Nature Science Foundation of China (grant nos. 82101672, 82171589), the National Key Research and Development Program of China (grant nos. 2022YFC2702504, 2019YFE0109500), the Basic and Applied Basic Research Foundation of Guangdong Province (grant no. 2021A1515010774), and the Guangzhou Municipal Science and Technology Project (grant nos. 202102010075, 2023A4J0578). The authors declare that they have no conflict of interest in relation to the data in this paper.

**TRIAL REGISTRATION NUMBER:**

N/A

## Introduction

Temporary ejaculation failure caused by excessive mental stress in males during IVF is an occasional problem that must be addressed. Despite interventions such as visual stimulation or partner assistance, or the oral administration of sildenafil, some men may still experience ejaculation failure. To circumvent the absence of sperm for fertilization on the day of oocyte retrieval, two primary strategies are employed in clinical practice. For patients who have concerns regarding ejaculation failure, sperm can be pre-frozen prior to the day of oocyte retrieval, with subsequent thawing and use for fertilization on the day of oocyte retrieval. Otherwise, these patients may undergo percutaneous testicular sperm aspiration (TESA) on the day of oocyte retrieval, followed by ICSI. In addition, for those without cryopreserved sperm, the couple may opt for cryopreservation of the female partner’s oocytes by vitrification as an alternative to TESA. Therefore, when another scheduled reattempt to obtain sperm is successful, this third option involves the thawing of frozen oocytes followed by ICSI. However, there is no comprehensive comparative analysis of the clinical efficacy among these three options.

There have been some reports regarding surgical sperm retrieval in patients with ejaculation failure (Watkins et al., 1996; Emery et al., 2004; Junsheng et al., 2013; Ozer et al., 2020). The clinical outcomes of TESA-ICSI are similar between patients with temporary ejaculation failure and patients with obstructive azoospermia (Junsheng et al., 2013); however, the outcomes after the use of TESA have not been compared to those after the use of pre-frozen sperm or oocyte freezing and subsequent thawing. Although the clinical outcomes after TESA are similar to that of natural ejaculation for those with non-azoospermic male infertility, TESA has been observed to reduce the fertilization rate for the couple (Lee et al., 2018; Kendall Rauchfuss et al., 2021) and potentially cause adverse effects, including bleeding, infection, pain, and decreased gonadal function in the man (Karacan et al., 2013). In contrast, the use of thawed pre-frozen sperm can achieve favorable fertilization rates and clinical outcomes (Torra-Massana et al., 2023). Oocyte cryopreservation with subsequent thawing can also be applicable to patients with ejaculation failure. Because of technological advancements, oocytes can now withstand the vitrification process and achieve good clinical outcomes after thawing (Solé et al., 2013; Cornet-Bartolomé et al., 2020). However, it is unclear whether the clinical outcomes of frozen–thawed oocytes are similar to those of fresh oocytes, when used with fresh testicular spermatozoa at a later date (Crawford et al., 2017; Eaton et al., 2020).

Therefore, we aimed to conduct a comparative analysis of the clinical outcomes associated with the use of pre-frozen sperm or oocyte cryopreservation versus TESA for patients experiencing ejaculation failure on the day of oocyte retrieval. The findings may serve to optimize patient management and improve the ART outcomes.

## Materials and methods

### Study population and design

Patients undergoing IVF treatment at the Reproductive Center of the Third Affiliated Hospital of Guangzhou Medical University between 1 January 2012 and 31 May 2021 were included in this study. Male patients with abnormal erectile function or obstructive/non-obstructive azoospermia requiring surgical sperm retrieval were excluded. All couples used autologous sperm and oocytes, while donor sperm and oocytes were excluded. If the male partner experienced ejaculation failure on the day of oocyte retrieval, the couple could use fresh TESA, thawed pre-frozen sperm, or oocyte freezing with subsequent thawing for treatment. This study was approved by the Regional Ethics Committee of our hospital, and all patients provided written informed consent.

### Ovarian stimulation and oocyte retrieval

Ovarian stimulation was performed using a GnRH agonist, a GnRH antagonist, or a mild-stimulation protocol. hCG (4000–10 000 IU) was administrated to induce final oocyte maturation when at least two follicles were >17 mm in diameter. Oocytes were collected 34–36 h after hCG injection.

### Methods and procedures for sperm retrieval

Spermatozoa were collected from patients through masturbation or TESA. Abstinence for 2–5 days was essential before collecting sperm through masturbation. On the day of sperm retrieval through masturbation, the hands and external genitalia of patients were washed to avoid contamination. In cases of ejaculation failure after masturbation, visual stimulation, partner assistance, or 100 mg sildenafil (Pfizer, Sandwich, UK) was used to improve erectile function and increase the success rate of semen retrieval.

If the patient was still unable to ejaculate after taking sildenafil and had no pre-frozen sperm available, TESA could be performed after obtaining written informed consent. A finger touch was used to identify the locus of the biopsy, and the patients were administered local anesthesia via injection of 5 ml 2% lidocaine. A biopsy was then performed using a 21-gauge butterfly needle connected to a 20-ml syringe. The biopsied sample was transferred to a culture dish containing the G-MOPS^TM^ PLUS medium (Vitrolife, Göteborg, Sweden), and spermatozoa were released using two fine forceps and observed under an inverted microscope (TE300; Nikon, Tokyo, Japan) at 400× magnification. When sufficient spermatozoa with normal morphology were obtained, the procedure was considered successful and terminated. If no spermatozoa were observed, a second biopsy was performed on the other testicle. Spermatozoa were collected by centrifuging the obtained solution in a Falcon tube (352003; Becton Dickinson, Franklin Lakes, NJ, USA) at 500 g for 10 min. Subsequently, ICSI was performed following a standard procedure (Esteves et al., 2018).

### Sperm freezing and thawing

Sperm from patients who had previously experienced ejaculation failure were frozen in advance. At room temperature, an equal amount of sperm freezing medium (SAGE, Trumbull, CT, USA) was added dropwise to the semen using a Pasteur pipette and gently shaken to thoroughly mix. The mixture was transferred into a cryotube (usually 1.5 ml/tube) and placed at 4°C for 30 min. It was then suspended 10 cm above liquid nitrogen for 30 min, and finally stored in liquid nitrogen. For thawing, the semen was removed from the liquid nitrogen and quickly placed in a 37°C water bath. After 5 min, the cryotube was removed from the water bath, and routine semen treatment was performed (Esteves et al., 2018).

### Fertilization via IVF or ICSI, and embryo culture and evaluation

Patients with thawed sperm underwent IVF and/or ICSI depending on the condition of the sperm, while patients with TESA or oocyte cryopreservation and subsequent thawing underwent ICSI. Fertilization was evaluated 16–18 h after routine fertilization or injection. The fertilized embryos were cultured separately in 25 µl of G-1^TM^ PLUS medium (Vitrolife) with Ovoil (Vitrolife) in a tri-gas incubator (5% O_2_/6% CO_2_/89% N_2_) at 37°C. On Day 3, the embryos were transferred to the G-2^TM^ PLUS medium (Vitrolife) for cultivation until Day 5/6.

Embryo quality was assessed on Days 3, 5, and 6. On Day 3, embryos with 7–9 blastomeres of uniform size and <20% fragmentation, and originating from two pronuclei, were classified as high-quality embryos; those with ≥5 blastomeres of uniform/relatively uniform size and <20% fragmentation were classified as usable embryos; and those with severely uneven size or >20% fragmentation were classified as abandoned embryos. Embryo quality was assessed at the blastocyst stage according to Gardner’s classification (Gardner et al., 2000). For blastocysts graded as 3–6 (i.e., full blastocysts onward), the development of the inner cell mass was assessed as follows: A, tightly packed, many cells; B, loosely grouped, several cells; or C, very few cells. The trophectoderm was assessed as follows: A, many cells forming a cohesive epithelium; B, few cells forming a loose epithelium; or C, very few large cells. When the expansion degree was Grade 3–6 and the inner cell mass and trophectoderm were not C, they were evaluated as high-quality blastocysts. When both were C, they were evaluated as abandoned blastocysts.

### Freezing and thawing of oocytes, embryos, and blastocysts

Oocytes, embryos, and blastocysts were frozen using vitrification reagent kits (Kitazato, Shizuoka, Japan). Briefly, oocytes were mixed with equal volumes of equilibration solution (ES) and G-MOPS^TM^ PLUS (Vitrolife) for 2 min at room temperature, followed by mixing with another ES droplet for 2 min, and then transferred to a separate ES droplet for 5 min. Finally, they were rinsed with vitrification solution (VS) for 90–120 s. Subsequently, the oocytes were loaded onto carriers and rapidly injected with liquid nitrogen. Cleavage embryos were placed in ES at room temperature for 5 min, and blastocysts were placed in ES at 37°C for 2 min and then transferred to VS for 45–60 s. Finally, the embryos/blastocysts were loaded onto carriers and immediately placed in liquid nitrogen. Frozen oocytes, embryos, and blastocysts were progressively thawed using different concentrations of sucrose. They were drained from the carrier into 1.0 mol/L sucrose, incubated at 37°C for 1 min, and sequentially transferred to 0.5, 0.25, or 0 mol/L sucrose medium for 3 min at room temperature. Finally, the thawed oocytes were transferred to G-IVF^TM^ PLUS medium (Vitrolife) and placed in an incubator (6% CO_2_ and 5% O_2_) at 37°C for 2 h, followed by ICSI. Meanwhile, the embryos and blastocysts were transferred to the G-2^TM^ PLUS medium and placed in an incubator (6% CO_2_ and 5% O_2_) at 37°C for 2 h, followed by transfer.

### Endometrium preparation for the thawing cycle

This step was performed for females undergoing hormone replacement therapy or a natural cycle. Patients were examined after menstruation to assess endometrial thickness and determine progesterone levels. When conditions were deemed appropriate (i.e. endometrium thickness ≥7 mm and progesterone level <1.5 ng/ml), cleavage embryo or blastocyst transfer was performed on Day 3/5 after ovulation or on Day 4/6 after progesterone exposure. All patients who underwent embryo transfer received progesterone as luteal support. During pregnancy, progesterone treatment was maintained at the same dose until 10 weeks of gestation. In cases of inadequate endometrial thickness or progesterone levels >1.5 ng/ml, embryo transfer was postponed to the subsequent cycle.

### Clinical outcome assessment

The primary outcome measure was the cumulative live birth rate (CLBR), while the secondary outcome measures were clinical pregnancy rate (CPR) per embryo transfer, and live birth rate (LBR) per embryo transfer. The CLBR was calculated as the ratio of the number of first deliveries (>24 weeks of gestation, including fresh and frozen cycles) with at least one live birth, to the number of retrieved oocyte cycles. The delivery of a singleton, twin, or other multiples is registered as one live birth. One completed cycle referred to a treatment cycle that achieved at least one live birth or a treatment cycle in which all embryos were transferred, but failed to achieve a live birth. The CLBR was computed with the completed cycles only. The CPR was calculated as the ratio of the number of clinical pregnancies with fetal heartbeat, to the number of embryo transfer cases, while the LBR was calculated as the ratio of the number of deliveries with at least one live birth, to the number of embryo transfer cases. In addition, we analyzed the rates of implantation, multiple pregnancies, and miscarriages, as well as laboratory outcomes.

### Statistical analyses

Statistical analyses were conducted using SPSS 22.0 (IBM, Armonk, NY, USA). Continuous data are expressed as medians (interquartile ranges [IQR]) and were compared using one-way analysis of variance. Categorical data are presented as percentages and were compared using the chi-square test. Kaplan–Meier curves were constructed with live birth as an event to illustrate the differences in CLBRs between different groups as the number of embryo transfers increased. A robust (modified) Poisson regression model was used to evaluate the association between the three clinical options for ejaculation failure and CLBRs. The variables in the regression model included the clinical options, female age at oocyte retrieval, type and duration of infertility, female BMI, number of previous IVF cycles, ovarian stimulation protocol, endometrial thickness on the last ultrasound, insemination method, number of oocytes retrieved, number of fertilized oocytes, and number of usable embryos on Day 3. For variables that significantly affect CLBR, receiver operating characteristic (ROC) curve and AUC were created to evaluate the predictive utility of the model. In addition, a modified Poisson regression model was also used to identify patient characteristics associated with the decision to use TESA rather than oocyte freezing for patients with ejaculation failure. These data were also adjusted for female age at oocyte retrieval, male age, number of IVF cycles, number of oocytes retrieved, endometrial thickness on the last ultrasound, and the presence of severe oligozoospermia (yes/no). Severe oligozoospermia was defined as a concentration of <5 million/ml. The risk ratio (RR), adjusted RR, and 95% CI were also calculated. Differences were considered statistically significant at *P* < 0.05.

A *post-hoc* power analysis was conducted using G*Power 3.1.9.7 (Franz Faul, Universität Kiel, Germany). With a two-sided alpha level of 0.05, the study’s sample size provided over 80% power to detect a 15.5% difference in CLBRs between TESA and frozen–thawed oocytes, as well as a 12% difference between TESA and pre-frozen sperm, which were set as thresholds for statistical significance.

## Results

**Figure 1.**
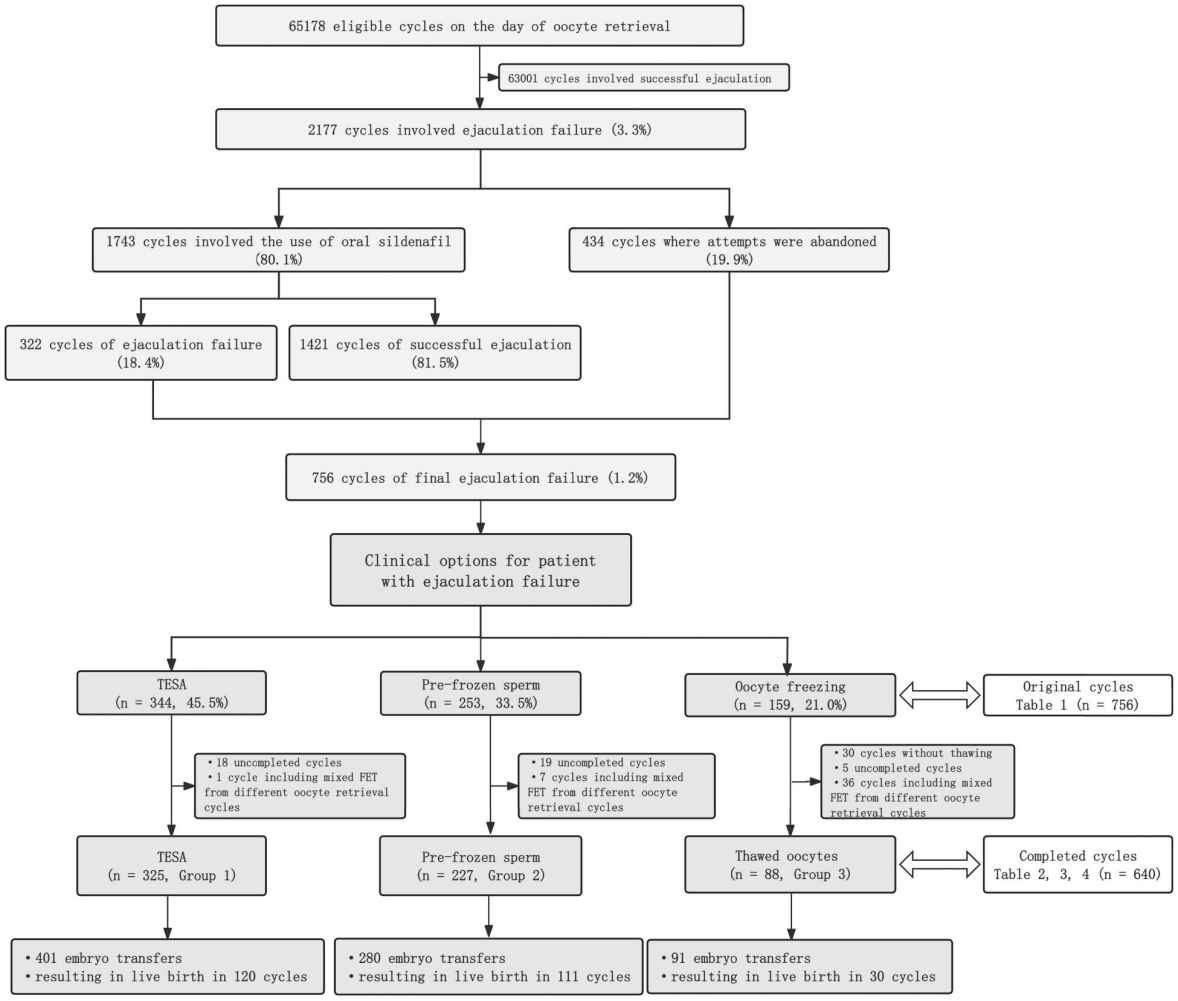
**Data selection flowchart.** This study analyzed the outcomes after three clinical options, namely, testicular sperm aspiration (TESA), the use of pre-frozen sperm, and oocyte freezing with subsequent thawing, for eligible couples undergoing IVF but who experienced ejaculation failure on the day of oocyte retrieval. Data were from the 10-year period from 2012 to 2021.

Out of 65 178 oocyte retrieval cycles (Fig. 1), 2177 cycles (3.3%) involved ejaculation failure. In this subgroup, 1743 cycles involved the use of oral sildenafil, with successful ejaculation achieved in 1421 cycles (81.5%). Ultimately, 676 patients in 756 cycles (1.2%) experienced ejaculation failure and underwent different clinical interventions, including TESA (n = 344), thawing of pre-frozen sperm (n = 253), or oocyte freezing (n = 159). Subsequently, 640 treatment cycles were completed: 325 cycles involved TESA (Group 1), 227 cycles involved pre-frozen sperm thawing (Group 2), and 88 cycles involved frozen-thawed oocytes (Group 3). Amongst these 640 completed cycles, a total of 772 embryo transfers were performed, resulting in live births in 261 cycles, including 120 cycles of TESA, 111 cycles with pre-frozen sperm thawing, and 30 cycles following oocyte thawing.

The characteristics of couples experiencing ejaculation failure are shown in Table 1. The female participants had a mean age of 35 years (IQR, 31–39), while their male counterparts had a mean age of 40 years (IQR, 35–45). The average duration of infertility for these couples was 4.25 years (IQR, 2–7). Respectively, male factors accounted for 20.8% (157/756), female factors accounted for 50.0% (378/756), and complex factors accounted for 29.2% (221/756) of the causes of infertility. Notably, approximately 65.8% (497/756) of these couples were undergoing their first IVF treatment.

**Table 1.** Characteristics of couples experiencing ejaculation failure, with the three clinical options.

Variable	All	TESA (control)	Pre-frozen sperm	Oocyte freezing	*P*-value
**Patient characteristics**					
Number of patients, n	676	321	238	149	
Number of cycles, n	756	344	253	159	
Female age at oocyte retrieval (years)	35 (31, 39)	35 (31, 38)	35 (30, 38)	36 (32, 40)*	0.016
Male age at oocyte retrieval (years)	40 (35, 45)	40 (36, 46)	39 (34, 44)*	40 (36, 46)	0.040
Duration of infertility (years)	4.25 (2, 7)	5 (3, 8)	4 (2, 7)	4 (2,7)	0.198
AMH (ng/ml)	2.6 (1.3, 5.3)	2.7 (1.5, 5.5)	2.6 (1.4, 5.2)	2.1 (0.8, 4.8)	0.372
FSH (IU/L)	5.6 (4.5, 7.1)	5.5 (4.4, 7.0)	5.6 (5.5, 7.2)	5.8 (4.5, 7.3)	0.087
Maternal BMI (kg/m^2^)	21.8 (20.2, 24.3)	22.5 (20.5, 25.1)	21.4 (19.8, 23.3)*	22.0 (20.0, 24.5)	0.001
Type of infertility, n (%)					0.186
Primary	338 (44.7%)	154 (44.8%)	122 (48.2%)	62 (39.0%)	
Secondary	418 (55.3%)	190 (55.2%)	131 (51.8%)	97 (61.0%)	
Main infertility causes, n (%)					0.270
Female factor	378 (50.0%)	173 (50.3%)	130 (51.4%)	75 (47.2%)	
Male factor	157 (20.8%)	81 (23.5%)	46 (18.2%)	30 (18.9%)	
Male and female factors	221 (29.2%)	90 (26.2%)	77 (30.4%)	54 (33.9%)	
Number of IVF cycles, n (%)					0.003
First cycle	497 (65.8%)	248 (72.1%)	154 (60.9%)*	95 (59.8%)*	
Second cycle	156 (20.6%)	65 (18.9%)	58 (22.9%)	33 (20.7%)	
≥ Third cycle	103 (13.6%)	31 (9.0%)	41 (16.2%)*	31 (19.5%)*	
**Semen analysis**					
Sperm concentration (million/ml)	48 (26, 82)	50 (27, 84)	42 (23, 78)	50 (27, 83)	0.127
Sperm motility (A + B) (%; IQR)	43 (25, 60)	41 (19, 61)	43 (27, 60)	45 (26, 56)	0.282
Severe oligozoospermia (<5 million/ml)	41 (5.4%)	12 (3.5%)	8 (3.2%)	21 (13.2%)*	0.000
**Characteristics of fresh cycles**					
Ovarian stimulation protocol, n (%)					0.000
GnRH agonist	376 (49.8%)	189 (55.0%)	125 (49.4%)	62 (39.0%)*	
GnRH antagonist	221 (29.2%)	113 (32.8%)	74 (29.3%)	34 (21.4%)*	
Mild-stimulation	159 (21.0%)	42 (12.2%)	54 (21.3%)*	63 (39.6%)*	
Started FSH dose (IU)	150 (150, 225)	187 (150, 225)	150 (150, 225)	150 (150, 225)*	0.000
Total FSH dose (IU)	1968 (1425, 2700)	2100 (1500, 2925)	1975 (1356, 2700)	1700 (1200, 2475)*	0.000
No. of days of ovarian stimulation	11 (9,12)	11 (9, 12)	11 (9, 12)	10 (8, 12)*	0.011
No. of oocytes retrieved	10 (5, 16)	10 (6,16)	9 (5,15)	8 (2,14)*	0.029

TESA, testicular sperm aspiration; AMH, anti-Müllerian hormone.

Continuous data are expressed as medians (interquartile ranges).

* *P* < 0 05, compared with TESA.

In 41 cycles, the male partner was diagnosed with severe oligozoospermia. For the remaining cycles, the average sperm concentration of the patients was 48 million/ml (IQR, 26–82 million/ml) and the average sperm motility was 43% (IQR, 25–60%). TESA treatment was used as the control group. Comparatively, the pre-frozen sperm group exhibited a significantly younger male age (*P* < 0.05), whereas the oocyte-freezing group demonstrated an older female age (*P* < 0.05). Both groups included a lower proportion undergoing their first IVF cycles and a higher proportion of mild-stimulation cycles (*P* < 0.01). The oocyte-freezing group included a higher proportion of cycles complicated by severe oligozoospermia (*P* < 0.001) and a lower number of retrieved oocytes (*P* < 0.05).

In the analysis of completed cycles (Table 2), TESA was used as the control group. In the comparisons, no significant differences were observed in the ages of either males or females at the time of oocyte retrieval, whether in the groups using pre-frozen sperm or thawed oocytes (*P* > 0.05). The group using pre-frozen sperm showed a significantly lower female BMI and a decreased proportion of women undergoing their first IVF cycle (*P* < 0.05), and 58.1% of these cycles achieved fertilization through IVF. The group using thawed oocytes showed a significantly reduced duration of infertility and lower female BMI. In the thawed-oocyte group, recurrent ejaculation failure occurred in 11 cycles (12.5%), with 6 cycles using pre-frozen sperm and 5 cycles using TESA. The survival rate of thawed oocytes was 87.2%. The thawed-oocyte group showed a significant reduction in the number of high-quality embryos and usable embryos on Day 3 (*P* < 0.05). In the embryo transfer cycles, no significant differences were found in the endometrial thickness, the developmental stage of the embryos, or the number of embryos transferred between the group undergoing TESA and the groups using pre-frozen sperm or frozen-thawed oocytes (*P* > 0.05). The CLBRs were 36.9%, 48.9%, and 34.1%, respectively. Compared with the TESA group, the thawed-sperm group showed a significant increase in CLBR (*P* = 0.007), but there were no significant differences in the CLBRs between the TESA group and the thawed-oocyte group (*P* > 0.05). The CPRs of the TESA, thawed-sperm, and thawed-oocyte groups were 37.4%, 50.0%, and 41.8%, respectively; the LBRs were 29.9%, 39.6%, and 33.0%, respectively; and the implantation rates were 28.7%, 37.9%, and 30.9%, respectively. Compared with the TESA group, the thawed-sperm group showed a significant increase in CPR (*P* = 0.005), LBR (*P* = 0.030), and implantation rate (*P* = 0.005), but no significant difference in multiple pregnancy rate (*P* > 0.05). In contrast, when compared with the TESA group, the thawed-oocyte group showed no significant differences in CPR, LBR, implantation rate, or multiple pregnancy rate (*P* > 0.05).

**Table 2.** Demographic and clinical characteristics of couples experiencing ejaculation failure and who completed treatment.

Variable	All groups	Group 1	Group 2	Group 3	*P*-value
		TESA (control)	Pre-frozen sperm	Frozen-thawed oocytes	
**Patient characteristics**					
Number of patients, n	584	303	218	88	
Number of oocytes retrieved cycles, n	640	325	227	88	
Female age at oocyte retrieval (years)	35 (31, 38)	35 (31, 38)	35 (30, 38)	34 (31, 39)	0.692
Male age at oocyte retrieval (years)	40 (35, 45)	40 (36, 46)	39 (34, 44)	39 (34, 46)	0.213
Duration of infertility (years)	5 (2, 7)	5 (3, 8)	4 (2, 7)	3.5 (2, 5)*	0.002
AMH (ng/ml)	2.7 (1.5, 5.4)	2.7 (1.5, 5.4)	2.6 (1.4, 5.1)	3.3 (1.9, 6.6)	0.321
FSH (IU/L)	5.6 (4.5, 7.0)	5.6 (4.5, 7.0)	5.6 (4.5, 7.2)	5.3 (4.3, 6.6)	0.107
Maternal BMI (kg/m^2^)	21.8 (20.2, 24.3)	22.5 (20.5, 25.1)	21.5 (19.9, 23.5)*	21.6 (19.7, 23.7)*	0.001
Type of infertility, n (%)					
Primary	289 (45.2%)	144 (44.3%)	108 (47.6%)	37 (42.1%)	0.614
Secondary	351 (54.8%)	181 (55.7%)	119 (52.4%)	51 (57.9%)	
Main infertility causes, n (%)					0.514
Female factor	323 (50.5%)	165 (50.8%)	117 (51.5%)	41 (46.6%)	
Male factor	139 (21.7%)	77 (23.7%)	42 (18.5%)	20 (22.7%)	
Male and female factors	178 (27.8%)	83 (25.5%)	68 (30.0%)	27 (30.7%)	
Number of IVF cycles, n (%)					0.039
First cycle	435 (68.0%)	233 (71.7%)	139 (61.2%)*	63 (71.6%)	
Second cycle	135 (21.1%)	63 (19.4%)	53 (23.4%)	19 (21.6%)	
≥ Third cycle	70 (10.9%)	29 (8.9%)	35 (15.4%)	6 (6.8%)	
**Semen analysis**					
Sperm concentration (million/ml)	48 (26, 82)	49 (27, 83)	43 (23, 79)	50 (27, 83)	0.175
Sperm motility (A + B) (%; IQR)	44 (26, 60)	42 (19, 61)	44 (27, 60)	46 (33, 59)	0.143
Severe oligozoospermia (< 5 million/ml)	24 (3.8%)	11 (3.4%)	6 (2.6%)	7 (8.0%)	0.118
**Characteristics of ovarian stimulation in fresh cycles**					
Ovarian stimulation protocol, n (%)					0.126
GnRH agonist	341 (53.3%)	177 (54.5%)	116 (51.1%)	48 (54.5%)	
GnRH antagonist	193 (30.2%)	106 (32.6%)	65 (28.6%)	22 (25.0%)	
Mild-stimulation	106 (16.5%)	42 (12.9%)	46 (20.3%)	18 (20.5%)	
Started FSH dose (IU)	150 (150, 225)	187.50 (150, 225)	150 (150, 225)	150 (150, 225)	0.095
Total FSH dose (IU)	2025 (1500, 2700)	2025 (1500, 2906)	2012 (1416, 2700)	1925 (1369, 2681)	0.197
No. of days of ovarian stimulation	11 (9, 12)	11 (9, 12)	11 (9, 12)	11 (9, 13)	0.649
No. of oocytes retrieved	10 (6, 16)	10 (6, 16)	9 (5, 15)	11 (6, 16)	0.141
**Laboratory data**					
Insemination methods					NA
IVF	132 (20.6%)	0 (0.0%)	132 (58.1%)*	0 (0.0%)	
ICSI	496 (77.5%)	325 (100.0%)	83 (36.6%)*	88 (100.0%)	
IVF + ICSI	12 (1.9%)	0 (0.0%)	12 (5.3%)*	0 (0.0%)	
Sperm types					NA
Fresh sperm	407 (63.6%)	325 (100.0%)	0 (0.0%)*	82 (93.2%)*	
frozen sperm	233 (36.4%)	0 (0.0%)	227 (100.0%)*	6 (6.8%)*	
Semen retrieval methods					NA
Ejaculate	310 (48.4%)	0(0.0%)	227 (100.0%)*	83 (94.3%)*	
TESA	330 (51.6%)	325 (100.0%)	0 (0.0%)*	5 (5.7%)*	
Survival rate of thawed oocytes (%)	NA	NA	NA	777/891 (87.2%)	NA
No. fertilized oocytes	6 (3, 10)	6 (3, 9)	6 (3, 10)	6 (4, 9)	0.430
No. two-pronucleate cleavage embryos	5 (3, 8)	5 (3, 8)	5 (2, 8)	5 (2, 8)	0.966
No. high-quality embryos on Day 3	1 (0, 2)	1 (0, 2)	1 (0, 2)	0 (0, 1)*	0.031
No. usable embryos on Day 3	2 (1, 5)	2 (1, 5)	3 (1, 5)	2 (1, 4)*	0.033
**Characteristics of endometrial preparation in FET cycles**					
Type of FET cycles, n (%)					0.684
Natural cycles	154 (29.7%)	65 (28.4%)	56 (32.2%)	33 (28.7%)	
Hormonal substitution cycles	364 (70.3%)	164 (71.6%)	118 (67.8%)	82 (71.3%)	
**Characteristics of total embryo transfer cycles**					
No. embryo transfer cycles, n	772	401	280	91	
Endometrial thickness of the last ultrasound (mm)	9.2 (8.0, 10.9)	9.4 (8.0, 11.0)	9.1 (8.0, 11.0)	9.0 (7.8, 10.0)	0.476
Development stage of embryo transferred, n (%)					0.147
Cleavage embryo	458 (59.3%)	245 (61.1%)	154 (55.0%)	59 (64.8%)	
Blastocyst	314 (40.7%)	156 (38.9%)	126 (45.0%)	32 (35.2%)	
Number of embryos transferred, n (%)					0.397
1	310 (40.2%)	170 (42.4%)	107 (38.2%)	33 (36.3%)	
≥2	462 (59.8%)	231 (57.6%)	173 (61.3%)	58 (63.7%)	
**Clinical outcomes per oocyte retrieval cycles**					
CLBR, n (%)	261 (40.8%)	120 (36.9%)	111 (48.9%)*	30 (34.1%)	0.007
**Clinical outcomes per embryo transfer cycles**					
CPR, n (%)	328 (42.5%)	150 (37.4%)	140 (50.0%)*	38 (41.8%)	0.005
LBR, n (%)	261 (33.8%)	120 (29.9%)	111 (39.6%)*	30 (33.0%)	0.030
Implantation rate (%)	402/1242 (32.4%)	183/637 (28.7%)	173/456 (37.9%)*	46/149 (30.9%)	0.005
Multiple pregnancy rate, n (%)	75 (22.9%)	33 (22.0%)	33 (23.6%)	9 (23.7%)	0.943
Miscarriage rate, n (%)	59 (18.0%)	26 (17.3%)	25 (17.9%)	8 (21.1%)	0.866

TESA, testicular sperm aspiration; AMH, anti-Müllerian hormone; FET, frozen embryo transfer; CPR, clinical pregnancy rate; LBR, live birth rate; CLBR, cumulative live birth rate.

Continuous data are expressed as medians (interquartile ranges).

* *P* < 0 05, compared with TESA.

**Figure 2.**
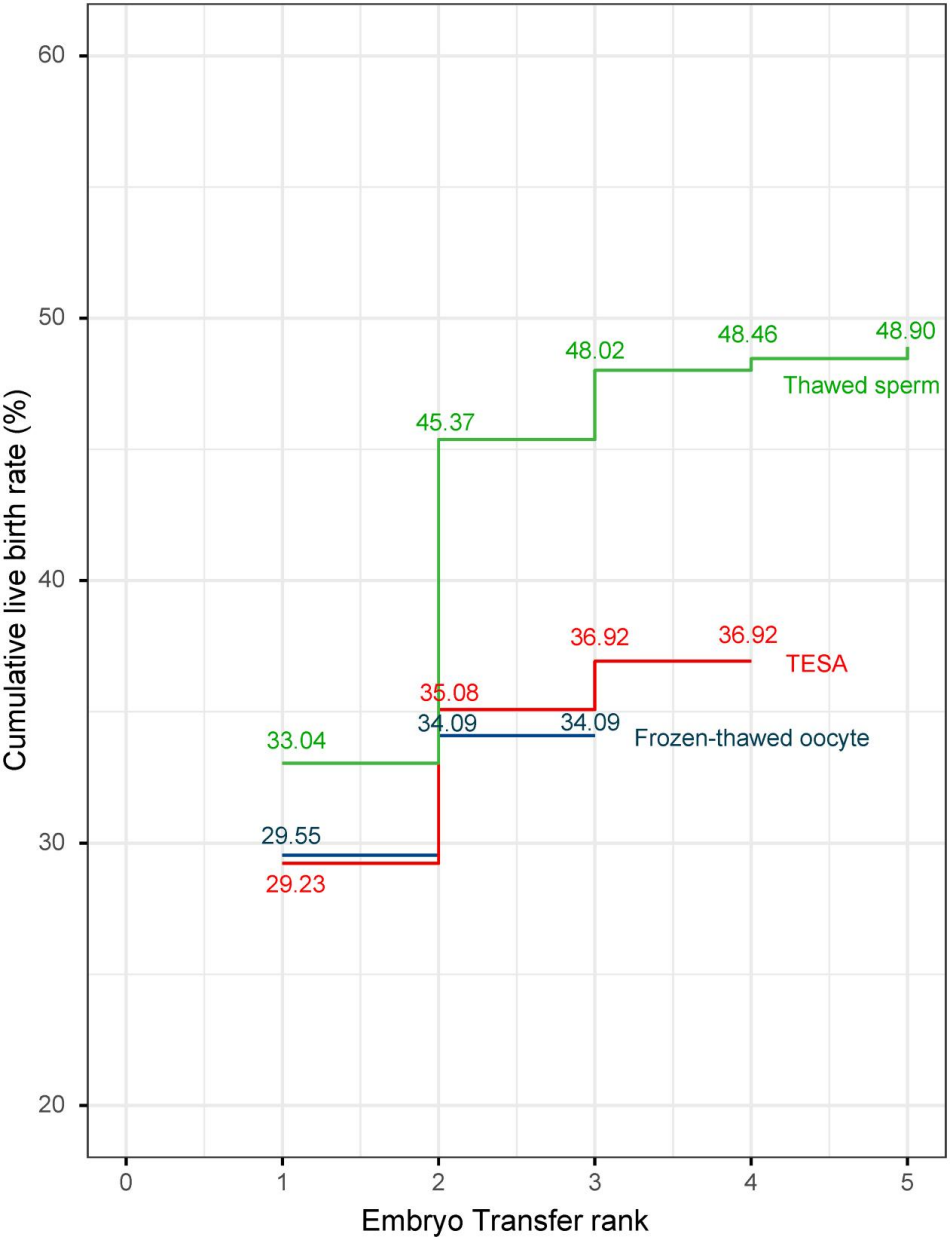
**Kaplan–Meier curves of cumulative live birth rates for couples who experienced ejaculation failure on the day of oocyte retrieval.** The curves show the cumulative live birth rates (CLBRs) for three clinical options as the number of embryo transfer cycles increased. Using the testicular sperm aspiration (TESA) group as the control group, after the second transfer, the use of pre-frozen sperm showed a significant increase in the CLBR (*P* < 0.05), while the thawed-oocyte group showed no significant difference (*P* > 0.05).

The CLBRs and LBRs of the three treatment groups based on the number of transfers are shown in Table 3. In total, the TESA group included 401 embryo transfer cycles (173 fresh + 228 frozen), the thawed-sperm group included 280 transfer cycles (106 fresh + 174 frozen), and the thawed-oocyte group included 91 transfer cycles (64 single-frozen + 27 double-frozen). Significant differences in both the CLBR and LBR were observed after the second transfer; the CLBR of the TESA, thawed-sperm, and thawed-oocyte groups were 35.1%, 45.4%, and 34.1%, respectively, while the corresponding LBR were 21.1%, 43.1%, and 25.0%, respectively. When compared with the TESA group, the thawed-sperm group showed a significant increase in both cumulative LBR (*P* = 0.033) and LBR (*P* = 0.012), but the thawed-oocyte group showed no significant differences in cumulative LBR or LBR (*P* > 0.05) (Table 3, Fig. 2).

**Table 3.** Calculation of LBRs and CLBRs for the completed cycles.

Embryo transfer	CLBR			*P*-value	LBR			*P*-value
	Group 1	Group 2	Group 3		Group 1	Group 2	Group 3	
	TESA (control, n = 325)	Pre-frozen sperm (n = 227)	Frozen–thawed oocytes (n = 88)		TESA (control)	Pre-frozen sperm	Frozen–thawed oocytes	
First	95 (29.2%)	75 (33.0%)	26 (29.6%)	0.616	95/285 (33.3%)	75/201 (37.3%)	26/73 (35.6%)	0.660
Second	114 (35.1%)	103 (45.4%)*	30 (34.1%)	0.033	19/90 (21.1%)	28/65 (43.1%)*	4/16 (25.0%)	0.012
Third	120 (36.9%)	109 (48.0%)*	30 (34.1%)	0.014	6/22 (27.3%)	6/11 (54.6%)	0/2 (0.0%)	0.131
Fourth	120 (36.9%)	110 (48.5%)*	30 (34.1%)	0.010	0/4 (0.0%)	1/2 (50.0%)	/	0.105
Fifth	120 (36.9%)	111 (48.9%)*	30 (34.1%)	0.007	/	1/1 (100.0%)	/	NA

LBR, live birth rate; CLBR, cumulative live birth rate; TESA, testicular sperm aspiration.

* *P* < 0 05, compared with TESA.

**Figure 3.**
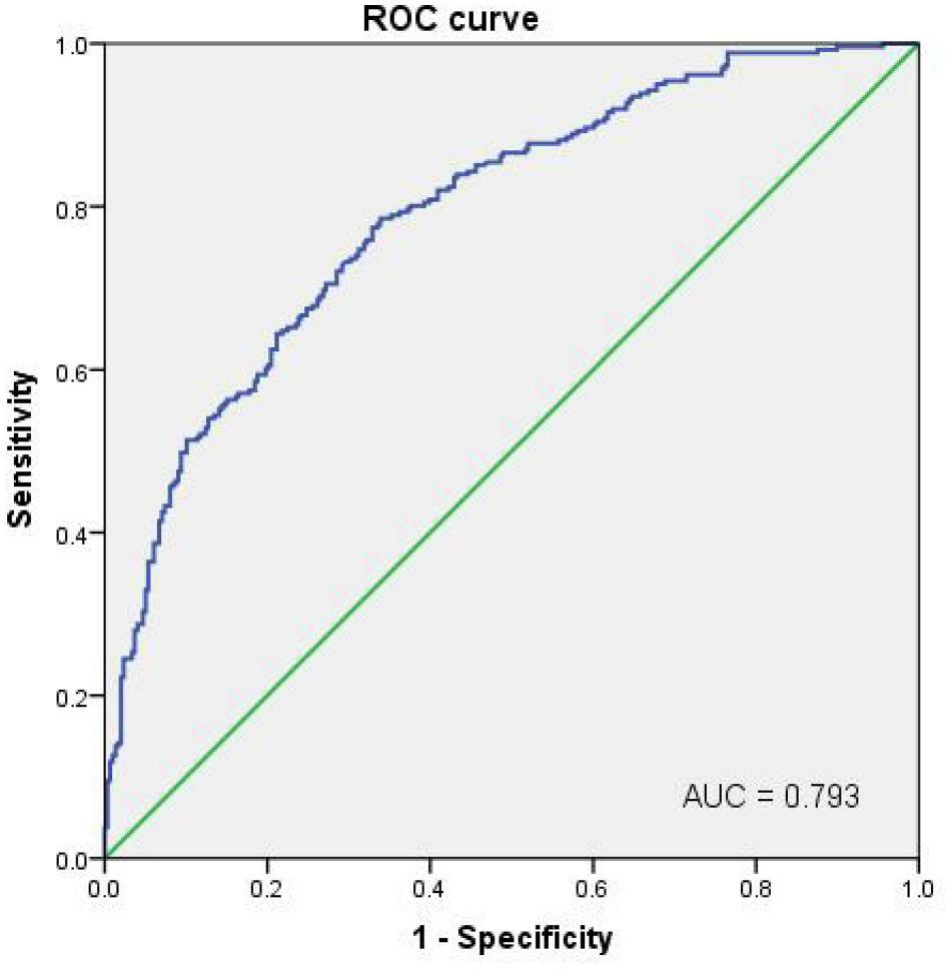
**Receiver operating characteristic (ROC) curve of the cumulative live birth prediction model for patients with ejaculation failure.** The predicted probability values were generated by three variables, including female age at oocyte retrieval, the number of usable embryos on Day 3, and endometrial thickness on the last ultrasound, were used to reach an area under the curve of 0.793.

A multivariable Poisson regression analysis of factors predicting the cumulative LBR of the oocyte retrieval cycle is shown in Table 4. The univariate analysis showed that using thawed sperm improved the cumulative LBR compared to TESA; however, after adjusting for multiple factors, no significant association was found between the cumulative LBR and the use of pre-frozen sperm (RR 1.08, 95% CI 0.81–1.44) or thawed oocytes (RR 1.01, 95% CI 0.76–1.33). Additionally, this model demonstrated that female age at oocyte retrieval (RR 0.95, 95% CI 0.93–0.97, *P* < 0.001), the number of usable embryos on Day 3 (RR 1.09, 95% CI 1.05–1.13, *P* < 0.001), and the endometrial thickness on the last ultrasound (RR 1.06, 95% CI 1.02–1.11, *P* = 0.005) were significantly associated with the cumulative LBR. The ROC curve for this model is plotted in Fig. 3, indicating an area under the curve of 0.793, with a specificity of 78.2% and a sensitivity of 64.8%.

**Table 4.** Regression analysis of factors predicting a cumulative live birth in an oocyte retrieval cycle.

Factors	Unadjusted		Adjusted	
	RR (95% CI)	*P*-value	RR (95% CI)	*P*-value
Clinical options				
Group 1: TESA	Ref.		Ref.	
Group 2: Pre-frozen sperm	1.31 (1.09–1.58)	0.004	1.08 (0.81–1.44)	0.599
Group 3: Thawed oocytes	0.98 (0.72–1.33)	0.877	1.01 (0.76–1.33)	0.965
Insemination methods				
IVF	Ref.		Ref.	
ICSI	0.70 (0.60–0.87)	0.001	0.83 (0.69–1.01)	0.052
IVF + ICSI	1.13 (0.73–1.73)	0.583	1.24 (0.79–1.95)	0.354
Ovarian stimulation protocol, n (%)				
GnRH agonist	Ref.		Ref.	
GnRH antagonist	1.05 (0.88–1.26)	0.601	1.07 (0.90–1.27)	0.452
Mild-stimulation	0.42 (0.27–0.66)	0.000	0.63 (0.39–1.02)	0.061
Number of IVF cycles, n (%)				
First cycle	Ref.		Ref.	
Second cycle	0.87 (0.69–1.09)	0.213	1.03 (0.82–1.29)	0.827
≥Third cycle	0.49 (0.31–0.80)	0.004	0.95 (0.58–1.55)	0.838
Female age at oocyte retrieval (years)	0.93 (0.92–0.95)	0.000	0.95 (0.93–0.97)	0.000
No. usable embryos on Day 3	1.10 (1.07–1.12)	0.000	1.09 (1.05–1.13)	0.000
Endometrial thickness of the last ultrasound (mm)	1.06 (1.02–1.11)	0.004	1.06 (1.02–1.11)	0.005

TESA, testicular sperm aspiration; RR, risk ratio.

RR: The variables in the regression model include clinical strategies, maternal age at oocyte retrieval, type of infertility, duration of infertility, maternal BMI, number of IVF cycles, ovarian stimulation protocol, endometrial thickness of the last ultrasound, insemination methods, number of oocytes retrieved, fertilized oocytes, and usable embryos on Day 3.

Additionally, we also constructed a multivariable regression model to identify patient characteristics associated with the clinical decision to use TESA rather than oocyte freezing for patients with ejaculation failure (Supplementary Table S1). According to this model, patients who did not exhibit severe oligozoospermia (RR 2.00, 95% CI 1.21–3.10, *P* = 0.002) and those with a thicker endometrium on the last ultrasound (RR 1.04, 95% CI 1.02–1.07, *P* < 0.001) were more likely to undergo TESA treatment.

## Discussion

### Principal findings

This study aimed to analyze the clinical outcomes after using pre-frozen sperm or oocyte freezing compared to TESA in patients with ejaculation failure on the day of oocyte retrieval. After adjusting for confounding factors, the use of pre-frozen sperm or frozen-thawed oocytes appears to be equally effective as TESA in achieving cumulative LBR.

### Results and research implications

The profound psychological and physiological stresses of infertility and its therapeutic interventions on patients can lead to difficulties in sperm retrieval in some male patients on the day of ovulation or oocyte retrieval. In our study, the incidence of ejaculation failure on the day of oocyte retrieval was 1.2%. To ensure successful sperm retrieval and smooth IVF procedures on the day of oocyte retrieval, our center provides outpatient consultations for male patients who are planning for or currently undergoing IVF. For patients with a history of sperm retrieval difficulties, sperm cryopreservation is recommended before the day of oocyte retrieval in case of no ejaculation or erectile dysfunction. This helps reduce patient stress on the day of oocyte retrieval and promotes successful sperm retrieval. However, for patients who have not successfully obtained sperm for pre-freezing or those who are confident in successful sperm retrieval but ultimately fail to ejaculate on the day of oocyte retrieval, they have to choose TESA or oocyte freezing with subsequent thawing as alternative treatments.

TESA-ICSI is an effective method for patients with ejaculation failure. The majority of patients in the TESA group obtained sperm by unilateral biopsy. Six of these patients (1.7%), who required bilateral testicular biopsy because no spermatozoa were found in their initial testicular tissue sample, showed severe oligozoospermia in semen analysis. The use of testicular sperm minimizes the generation of high levels of reactive oxygen species during sperm passage via the seminiferous tubules and epididymis, thereby reducing sperm DNA fragmentation (Muratori et al., 2015; Cho et al., 2016). Therefore, TESA-ICSI improves the outcomes for individuals with higher sperm DNA fragmentation (Bradley et al., 2016; Esteves et al., 2017). However, for other male patients, including those with oligozoospermia, TESA does not improve ICSI outcomes and is associated with lower fertilization and blastocyst formation rates (Kendall Rauchfuss et al., 2021). Other studies have shown that TESA-ICSI significantly reduces the fertilization rate compared to ejaculated sperm, but the potential for embryo and blastocyst formation is similar (Lee et al., 2018). At present, there has been no comparison between fresh sperm from TESA and frozen sperm from ejaculation. Our findings indicate no significant differences in fertilization and embryo development rates between patients undergoing TESA and those using thawed ejaculated sperm. Previous studies on sperm freezing have mainly focused on ICSI (Punjani et al., 2022; Torra-Massana et al., 2023), while our study included both IVF and ICSI to comprehensively evaluate the effects of pre-frozen sperm on fertilization and pregnancy outcomes in patients with ejaculation failure. Our study included patients with both normal and poor sperm quality. The freezing and thawing process may negatively impact sperm motility and the fertilization rate (Hammadeh et al., 1999; Borges et al., 2007; Kopeika et al., 2015). Previous studies have shown that, in males with normal sperm concentrations, the use of thawed sperm achieves fertilization, implantation, and CPRs similar to those achieved when using fresh sperm (Punjani et al., 2022; Torra-Massana et al., 2023). This may be because the sperm that survive after thawing are more robust and of high quality. Therefore, these patients undergo conventional IVF rather than ICSI. For patients with poor sperm quality, ICSI can overcome fertilization barriers. We found that the frozen-thawed sperm group had a CLBR higher than the TESA group by more than 10%. Further analysis showed that using pre-frozen sperm resulted in higher LBRs after the second transfer and an increased number of embryo transfers, ultimately increasing the CLBR. This may be attributed to the availability of more high-quality embryos with normal morphology or ploidy. Therefore, this study suggests that pre-frozen sperm may be conducive to enhance the CLBR in patients with ejaculation failure, even though no statistically significant association was found between pre-frozen sperm and CLBR after adjusting for confounding factors. However, since the occurrence of ejaculation failure is difficult to predict accurately for both patients and urologists, recommending routine sperm cryopreservation for all IVF candidates is impractical. Given that TESA is an invasive procedure with potential complications (Esteves et al., 2013), cryopreservation of sperm before oocyte retrieval emerges as a non-invasive and effective method for patients with concerns of temporary ejaculation failure. Meanwhile, for patients experiencing ejaculation failure without pre-frozen sperm, TESA remains a viable and effective option to complete their IVF cycles.

Oocyte freezing is another remedial intervention for patients with ejaculation failure on the day of oocyte retrieval. Within our study cohort, of those experiencing ejaculation failure who chose oocyte freezing, 30 couples (18.87%) had their oocytes retained in storage, possibly due to recurrent ejaculation failure in the male partner and concerns about the risks associated with the TESA procedure, or worsening anxiety resulting in cancellation of the sperm extraction surgery. In addition, some patients may have gone to other fertility centers to restart IVF treatment. Among the patients who completed the cycles with frozen–thawed oocytes, 11 (12.5%) chose to freeze sperm in advance or undergo TESA to obtain sperm, while the remaining patients successfully obtained ejaculated sperm for ICSI. The fertilization rate and number of high-quality embryos are similar after the use of frozen–thawed oocytes or fresh oocytes (Rienzi et al., 2010; Solé et al., 2013). However, some studies have shown that frozen oocytes produce lower fertilization rates and embryo morphology scores than fresh oocytes (Cornet-Bartolomé et al., 2020; Melado et al., 2020; Fu et al., 2021). In this study, the TESA group used fresh oocytes combined with surgically retrieved fresh sperm, while the thawed oocytes group mostly used frozen-thawed oocytes combined with fresh ejaculates, with a small number of cases using frozen-thawed oocytes combined with fresh surgically retrieved sperm or frozen-thawed ejaculates. We observed that, in contrast to the TESA group using fresh oocytes, the thawed-oocyte group had a significantly lower number of high-quality embryos and usable embryos on Day 3, which may be mainly related to the adverse effects of the freezing and thawing processes. Although the survival rate of frozen oocytes has been improved with the development of vitrification technology, the thawing process can inevitably cause damage or even induce degeneration in some oocytes, thereby compromising subsequent embryonic development. Previous studies have shown that there is no significant difference in CPR or LBR between embryos developed from vitrified oocytes and those from fresh oocytes (Solé et al., 2013; Cornet-Bartolomé et al., 2020). Our research showed no difference in CPR or LBR per embryo transfer between patients undergoing ICSI with thawed oocytes and those undergoing TESA-ICSI with fresh oocytes. A multiple regression analysis also showed no statistically significant difference in CLBRs between these two groups. This is inconsistent with a previous study showing differences in cumulative outcomes between cycles using cryopreserved and fresh oocytes (Crawford et al., 2017), possibly due to disparate sperm sources between these two studies. Our findings suggest that, for patients experiencing ejaculation failure in the absence of pre-frozen sperm, both TESA and oocyte freezing are effective strategies, as they show comparable CLBRs. However, it is unclear whether the use of thawed embryos from vitrified oocytes that have undergone two rounds of vitrification will have long-term effects on newborns, warranting more in-depth studies (Cobo et al., 2013).

For patients experiencing ejaculation failure without pre-frozen sperm, a variety of clinical and demographic variables may affect the clinical intervention option. Therefore, we evaluated the characteristics of patients with ejaculation failure who chose TESA instead of oocyte freezing. We found that neither the age of the males and females nor the number of retrieved oocytes affected the clinical options. However, the absence of severe oligozoospermia in males and a thicker endometrium in females showed a positive association with TESA selection, possibly contributing to a faster embryo transfer. Due to problems associated with a fresh embryo transfer, patients with a thinner endometrium may chose oocyte freezing instead of embryo freezing. In addition, men with severe oligozoospermia may also choose oocyte freezing due to concerns about insufficient spermatozoa, and may consider freezing ejaculated sperm in advance for subsequent treatment.

The strength of the current study is that it is the first comprehensive analysis of the outcomes associated with three different clinical strategies for patients with ejaculation failure in the same institution over a decade-long period. The findings provide valuable clinical guidance for this patient cohort. However, our study also has some limitations. First, despite the inclusion of a sample size of 10 years, due to the rarity of this acute condition, we observed a relatively small number of samples with ejaculation failure, particularly concerning oocyte freezing strategy. Therefore, the power to detect differences in CLBRs among the three options is limited. Second, the retrospective nature of the study introduces a potential of selection bias. Although the regression model suggests no association between clinical options and the primary endpoint, the nominal CLBR favored pre-frozen sperm. Therefore, further research in the form of prospective as well as randomized controlled trials is needed to provide a definitive answer to the research question. Third, due to the small number of newborns from single pregnancies after fresh and thawed embryo transfer, we were unable to further analyze the neonatal outcomes of these three clinical options.

## Conclusion

In conclusion, patients experiencing ejaculation failure on the day of oocyte retrieval can benefited from three clinical options, namely TESA, the use of pre-frozen sperm, or the option of oocyte freezing with thawing at a later date when sperm is available. Our findings suggest that the use of pre-frozen sperm or thawed oocytes appear to be as effective as TESA in achieving CLBRs in patients with temporary ejaculation failure.

## Supplementary Material

deae290_Supplementary_Table_S1

## Data Availability

The data underlying this article will be shared upon reasonable request to the corresponding author.
