# Supplementary material for: Comparison of fresh testicular sperm aspiration and use of either thawed pre-frozen sperm or oocyte freezing: impact on cumulative live birth rates for couples experiencing ejaculation failure
Source: Hum Reprod. 2024 Dec 31;40(2):199–209. doi: 10.1093/humrep/deae290 (PMC11788192; doi:10.1093/humrep/deae290)
Supplement: deae290_Supplementary_Table_S1 [file deae290_supplementary_table_s1.pdf]

Human Reproduction, 2024, 1–1
https://doi.org/10.1093/humrep/deae290
Supplementary Data

Supplementary Table S1. Multivariable regression model to identify patient characteristics associated with the decision to use TESA rather than oocyte freezing for couples with ejaculation failure.

Factors	Adjusted RR (95% CI)	P-value
Female age at oocyte retrieval (years)	0.99 (0.98–1.01)	0.267
Male age at oocyte retrieval (years)	1.01 (0.99–1.02)	0.265
Severe oligozoospermia		
Yes	Ref.	
No	2.00 (1.21–3.10)	0.002
Number of IVF cycles, n (%)		
First cycle	Ref.	
Second cycle	0.94 (0.81–1.09)	0.419
≥Third cycle	0.80 (0.62–1.04)	0.092
Endometrial thickness of the last ultrasound (mm)	1.04 (1.02–1.07)	< 0.001
No. of oocytes retrieved	1.01 (0.99–1.01)	0.678

RR, risk ratio.
A total of 503 cycles of TESA and oocyte freezing were analyzed.
RR: adjusted for maternal age at oocyte retrieval, paternal age, number of IVF cycles, number of oocytes retrieved, endometrial thickness of the last ultrasound, severe oligozoospermia (yes/no).

© The Author(s) 2024. Published by Oxford University Press on behalf of European Society of Human Reproduction and Embryology.
This is an Open Access article distributed under the terms of the Creative Commons Attribution License (https://creativecommons.org/licenses/by/4.0/), which permits unrestricted reuse, distribution, and reproduction in any medium, provided the original work is properly cited.
Human Reproduction, 2024, 00, 1–11
https://doi.org/10.1093/humrep/deae290
Original Article
